# Infant Foods

**Published:** 1890-09

**Authors:** 


### INFANT FOODS.

At a meeting of the American Association for the Advancement of Science, Dr. Frederick Hoffman spoke about the food preparations now so largely used for infants and children as substitutes for mother milk and other food. According to reliable estimates, the quantity of such artificial food annually consumed in the United States amounts, in money value, to about $8,000,000 to $10,000,000. The problem of artificial food for the rising generation, and particularly during the first period of its struggle for life and survival, is a very important one, not only to the state, the commonwealth and the family, but also to the sanitarian and the chemist. These artificial foods mostly claim to be perfect ; a few have been analyzed by independent chemists, often with questionable accuracy or results, whilst many are of unknown composition. Many of such food preparations are evidently manufactured without due consideration to physiological and chemical principles in regard to the proper constituents and proportions of the true nutrient elements of food. Any such deficiencies or worthlessness are of much greater harm than similar short-comings in foods and food luxuries of the adult population.

